# Topological analysis of 3D digital ovules identifies cellular patterns associated with ovule shape diversity

**DOI:** 10.1242/dev.202590

**Published:** 2024-05-30

**Authors:** Tejasvinee Atul Mody, Alexander Rolle, Nico Stucki, Fabian Roll, Ulrich Bauer, Kay Schneitz

**Affiliations:** ^1^ Plant Developmental Biology, TUM School of Life Sciences, Technical University of Munich, Emil-Ramann-Strasse 4, 85354 Freising, Germany; ^2^ Applied and Computational Topology, TUM School of Computation, Information and Technology, Technical University of Munich, Boltzmannstrasse 3, 85747 Garching, Germany; ^3^ Munich Data Science Institute, Technical University of Munich, Walther-von-Dyck Strasse 10, 85747 Garching, Germany

**Keywords:** 3D digital organ, Ovule, Cardamine, *Arabidopsis*, Morphogenesis, Evo-devo, Topology

## Abstract

Tissue morphogenesis remains poorly understood. In plants, a central problem is how the 3D cellular architecture of a developing organ contributes to its final shape. We address this question through a comparative analysis of ovule morphogenesis, taking advantage of the diversity in ovule shape across angiosperms. Here, we provide a 3D digital atlas of *Cardamine hirsuta* ovule development at single cell resolution and compare it with an equivalent atlas of *Arabidopsis thaliana*. We introduce nerve-based topological analysis as a tool for unbiased detection of differences in cellular architectures and corroborate identified topological differences between two homologous tissues by comparative morphometrics and visual inspection. We find that differences in topology, cell volume variation and tissue growth patterns in the sheet-like integuments and the bulbous chalaza are associated with differences in ovule curvature. In contrast, the radialized conical ovule primordia and nucelli exhibit similar shapes, despite differences in internal cellular topology and tissue growth patterns. Our results support the notion that the structural organization of a tissue is associated with its susceptibility to shape changes during evolutionary shifts in 3D cellular architecture.

## Teaser

**Summary:** A combination of topological analysis and quantitative morphometrics of 3D digital ovules at cellular resolution reveals shape-relevant diversity in 3D cellular architecture between ovules of *Cardamine hirsuta* and *Arabidopsis thaliana*.

## INTRODUCTION

How an organ achieves its size and shape remains a major unresolved biological question. In particular, little is known about the complex cellular behaviors that often lead to emergent tissue properties and ultimately to the functional architectures that make up a tissue or organ, although impressive progress has been made in dissecting the cellular basis of, for example, epithelial morphogenesis (Gómez-Gálvez et al., 2021; Lemke and Nelson, 2021; Sozen et al., 2022).

Plants are uniquely suited to study the cellular basis of tissue morphogenesis. Plant species are extremely diverse in size and shape, but are composed of relatively few cell types and tissues made up of immobile cells (Lyndon, 1990). To understand the differences in the 3D cellular architectures that underlie differences in morphogenesis, it is paramount to decipher the 3D cellular architecture of tissues at different developmental stages. Advances in 3D confocal imaging of fixed, cleared and stained organs, along with the development of open-source software for image processing, including 3D segmentation and mesh generation, have enabled 3D analysis at the cellular, tissue and organ levels (Barbier de Reuille et al., 2015; Kurihara et al., 2015; Musielak et al., 2015; Strauss et al., 2019, 2022; Tofanelli et al., 2019; Truernit et al., 2008). These advances allowed digital single cell analyses of organs with regular internal cellular architecture and simple shape, including early embryo, hypocotyl or root (Bassel et al., 2014; Duran-Nebreda et al., 2023; Fridman et al., 2021; Graeff et al., 2021; Hernandez-Lagana et al., 2021; Lora et al., 2017; Montenegro-Johnson et al., 2015; Ouedraogo et al., 2023; Pasternak et al., 2017; Schmidt et al., 2014; Vijayan et al., 2021; Yoshida et al., 2014).

Most of these 3D digital single cell analyses have focused on organs of *Arabidopsis thaliana* (*A. thaliana*). Comparative studies can explore the evolution of cellular diversity underlying morphogenesis across species and possibly result in the emergence of a finite set of formal rules that enable cell collectives to organize into functional architectures. However, a deep understanding of the 3D cellular basis of morphogenesis in different plant species is presently not available and is urgently needed.

Ovules are elaborately shaped organs that play a central role in female sexual reproduction in seed plants. At maturity, they carry the embryo sac with the actual egg cell enclosed by one or two integuments that protect the embryo sac and later develop into the seed coat. Several characteristics underlie ovule diversity among plant species, including ovule size, integument number and curvature (Endress, 2011). Ovule morphology has been extensively studied across a broad variety of plant species (Endress, 2011; Gasser and Skinner, 2019). However, a comprehensive quantitative understanding of the 3D cellular architecture underlying the differences in ovule morphology between species is still lacking.

Recently, a reference 3D digital atlas of *A. thaliana* ovule development with full single cell resolution was established (Vijayan et al., 2021). The atlas enabled quantitative analysis of 3D cell and tissue growth patterns and dynamics even for an organ with an intricate internal cellular architecture and complex overall shape. It provides a valuable reference for a detailed 3D comparative analysis of ovule development. *Cardamine hirsuta* (*C. hirsuta*) represents an exciting model system for comparative analysis of plant morphogenesis (Hay and Tsiantis, 2016; Hay et al., 2014). The species displays abundant morphological diversity compared with its relative *A. thaliana*, providing a solid basis for investigating the evolutionary basis of morphological diversity (Hay and Tsiantis, 2006; Kierzkowski et al., 2019; Vlad et al., 2014). In terms of ovule morphology, *C. hirsuta* and *A. thaliana* show differences in the shape of mature pre-fertilization ovules, as well as in ovule size, seed weight, size and shape (Hay et al., 2014; Neumann and Hay, 2020).

Here, we present a deep imaging-based reference 3D digital atlas of *C. hirsuta* ovule development with full single cell resolution. Furthermore, we combined a mathematical analysis of the topology of the 3D cellular architectures of *C. hirsuta* and *A. thaliana* ovules with comparative morphometrics. Using this interdisciplinary strategy, we identified and quantified divergent cellular patterns that may be associated with changes in ovule size and shape between the two species.

## RESULTS

### Generation of a reference 3D digital atlas of *C. hirsut*a ovule development with cellular resolution

**Fig. 1.**
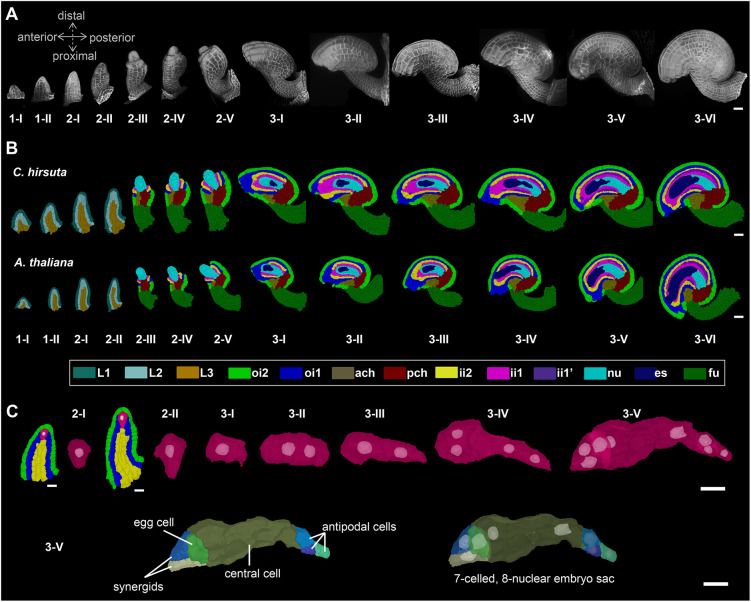
**A 3D digital atlas of *C. hirsuta* ovule development.** (A) *C. hirsuta* ovule development from initiation at stage 1-I to maturity at stage 3-VI. Images are 3D renderings of CLSM *z*-stacks of ovules with SR2200-stained cell walls. The anterior-posterior and proximal-distal axes are indicated. (B) Approximate mid-sagittal 2D sections of cell type-labeled 3D ovule meshes from stages 1-I to 3-VI showing the cell type organization in wild-type ovules. In *C. hirsuta*, stages 1-I to 2-II labeling includes radially organized L1, L2 and L3 layers. From stage 2-III up to 3-VI, individual cell-type labels are tissue specific and are represented with different colors. For comparison, an equivalent series is shown for *A. thaliana* (data obtained from Vijayan et al., 2021). (C) Megaspore mother cell development in *C. hirsuta* stage 2 primordia and embryo sac development from mono-nuclear embryo sac (stage 3-I) up to seven-celled, eight-nucleate embryo sac (stage 3-VI). ach, anterior chalaza; es, embryo sac; fu, funiculus; ii1, inner layer of inner integument; ii1′, parenchymatic layer of inner integument; ii2, outer layer of inner integument; nu, nucellus; oi1, inner layer of outer integument; oi2, outer layer of outer integument; pch, posterior chalaza. Scale bars: 20 µm in A and B; 10 µm in C.

To generate a comprehensive 3D digital atlas of *C. hirsuta* ovule development, we followed the procedure outlined previously (Tofanelli et al., 2019; Vijayan et al., 2021) and in the Materials and Methods section. In short, we performed 3D confocal laser scanning microscopy of fixed, cleared (Kurihara et al., 2015) and stained ovules to obtain z-stacks of optical sections of ovules of all stages (staging according to Schneitz et al., 1995; Vijayan et al., 2021). Ovules were stained with SR2200 to mark the cell outlines and TO-PRO-3 to label the nuclei (Bink et al., 2001; Van Hooijdonk et al., 1994). This was followed by cell boundary prediction and 3D cell segmentation using the PlantSeg pipeline (Wolny et al., 2020). The segmented stacks were then loaded into MorphoGraphX (MGX) software for mesh generation, cell type labeling and quantitative analysis. The entire procedure, from imaging to the final segmented and labeled 3D digital ovule, takes ∼2 h per *z*-stack. It is about twice the time required for *A. thaliana* ovules because *C. hirsuta* ovules are considerably larger in size. Overall, these efforts resulted in the generation of a high quality reference set of 144 hand-curated 3D digital ovules of the wild-type Oxford accession of *C. hirsuta* (≥10 samples per stage, 13 stages from stage 1-I to 3-VI) (Fig. 1).

The regions and types of segmentation errors in the cell-segmented stacks of *C. hirsuta* ovules are similar to those observed in the case of *A. thaliana* ovule segmentation (Tofanelli et al., 2019; Vijayan et al., 2021)*.* The main sources of error include two groups of cells: the megaspore mother cell (MMC) and its immediate lateral neighbors at stages 2-II to 2-V form the first group; and the embryo sac cells at stages 3-V and 3-VI form the second group. The main reasons for these errors could be insufficient cell wall staining with SR2200 or partially formed cell walls (Mansfield et al., 1991). Oversegmentation errors, if present, were corrected manually. Stage 1-I to 2-I and 3-I to 3-IV ovules selected for quantitative analysis had no apparent segmentation errors. Stage 2-II to 2-V ovules selected contained no more than five undersegmented (uncorrected) cells in the region occupied by the MMC and its lateral neighbors. Stage 3-V to 3-VI mature ovules selected had no obvious segmentation errors in the sporophytic tissue.

### General description of *C. hirsuta* ovule development

The ovules of *C. hirsuta* exhibit an overall tissue organization similar to that of the ovules of *A. thaliana* (Fig. 1A-C) (Robinson-Beers et al., 1992; Schneitz et al., 1995; Vijayan et al., 2021). To stage *C. hirsuta* ovule development, we adopted the staging system of *A. thaliana* (Schneitz et al., 1995; Vijayan et al., 2021). During stage 1, *C. hirsuta* ovule primordia emerge from the placenta as finger-like protrusions. During stage 2, three pattern elements can be distinguished along the proximal-distal axis: the proximal funiculus, which connects the ovule to the placenta; the central chalaza, from which an inner and outer integument emerge laterally; and the distal nucellus. The nucellus produces the large subepidermal MMC, which in turn undergoes meiosis during stages 2-IV and 2-V, with only one meiotic product developing into the haploid multicellular female gametophyte or embryo sac of the standard polygonum type during stage 3 (Fig. 1C). For most of its development, each integument is composed of two cell layers, an inner or adaxial layer and an outer or abaxial layer (Fig. 1A,B). In a sagittal section, each of these layers has a single cell thickness. At stage 2-II, development of the inner integument begins in a ring-like fashion and ends as a tube, whereas early development of the outer integument at stage 2-III is centered on the posterior side of the chalaza, but continues laterally to partially encircle the chalaza forming a hood-like structure. During stage 3, the two integuments grow asymmetrically over the nucellus, and eventually the ovule exhibits a pronounced curvature. The inner integument eventually generates a third layer. At their distal end, the integuments form a cleft, the micropyle, through which the pollen tube reaches the embryo sac containing the egg cell to affect fertilization. The position of the micropyle therefore influences pollen tube entry and, hence, fertilization.

### Overall size and appearance of *C. hirsuta* and *A. thaliana* ovules

**Fig. 2.**
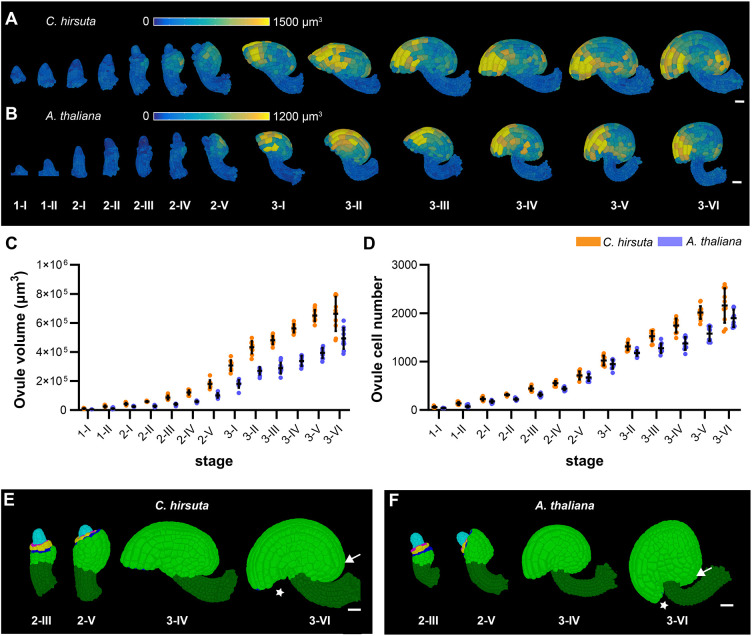
**Differences in ovule size and shape between *C. hirsuta* and *A. thaliana* ovules.** Comparison of ovule volume, cell number and ovule shape across stages of ovule development in *C. hirsuta* and *A. thaliana*. *A. thalian*a data are from Vijayan et al. (2021). (A,B) 3D cell meshes of the developmental series of wild-type ovules, showing heat maps of the cell volume of the outermost layer of *C. hirsuta* and *A. thaliana*. (C,D) Plots depicting the total volume and total number of cells of individual ovules from early to late stages of development, respectively. Data points indicate individual ovules. Data are mean±s.d. (E,F) Shape comparison. 3D cell meshes of wild-type ovules with colors indicating different tissues, as in Fig. 1B. Note the difference in positioning of the micropyle (stars) and the degree of posterior rounding (arrows). Scale bars: 20 µm.

To investigate the similarities and diversity of 3D cellular architectures underlying ovule development between *C. hirsuta* and *A. thaliana*, we performed a quantitative comparative study of the 3D digital ovule datasets. We first asked how much the ovule size of the two species differs and what the cellular basis of the difference is. We quantified the ovule size of *C. hirsuta* in terms of total volume and cell number, and found an incremental increase in both volume and cell number for each successive stage of development (Fig. 2A-D, Table 1). At stage 3-VI, the average cell number per *C. hirsuta* ovule is about 2160 cells and the mean volume per ovule is about 6.6×10^5^ μm^3^. By contrast, the *A. thaliana* ovule at stage 3-VI features about 1900 cells and a volume of about 5×10^5^ μm^3^. Thus, the stage 3-VI *C. hirsuta* ovule is ∼34% larger than the *A. thaliana* ovule and has 14% more cells. At all other developmental stages, *C. hirsuta* ovules are also significantly larger in volume and have somewhat higher cell numbers and cell volumes than corresponding *A. thaliana* ovules (Table 1). In conclusion, the increase in ovule size in *C. hirsuta* compared with *A. thaliana* is mainly due to a larger average cell size, with a smaller contribution from cell number.

**Table 1.**
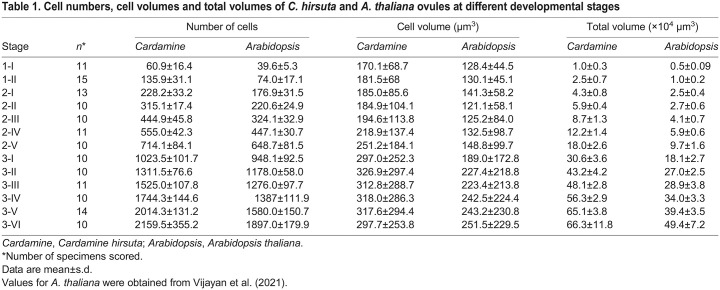
Cell numbers, cell volumes and total volumes of *C. hirsuta* and *A. thaliana* ovules at different developmental stages

Next, we compared the shape of the ovules (Fig. 2E,F). We found noticeable differences in curvature starting around stage 3-IV. We observed that the curvature of the ovule in *C. hirsuta* was not as advanced at the anterior side and the ovule appeared slightly more rounded at the posterior side compared with *A. thaliana*. Hence, the ovules of the two species ultimately differ in shape.

### Topological analysis reveals differences in 3D cellular architecture between *C. hirsuta* and *A. thaliana* ovules

**Fig. 3.**
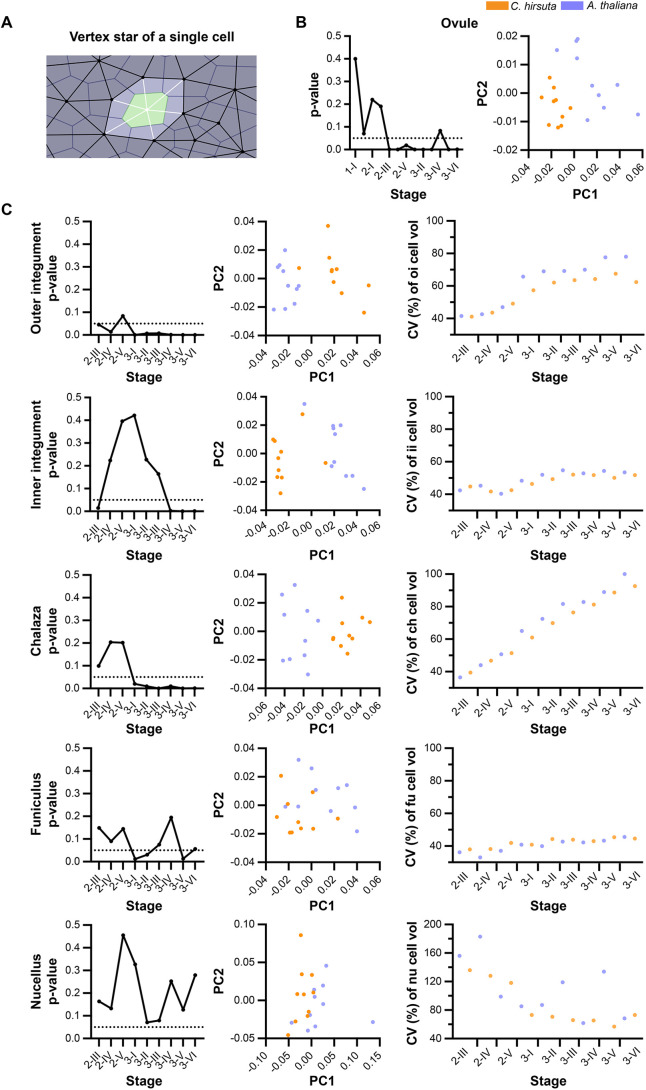
**Topological analysis of 3D cellular architectures for *C. hirsuta* and *A. thaliana*.** (A) The nerve complex (the triangles, with their edges and vertices) of a collection of cells. The 0-, 1- and 2-simplices of the vertex star (one vertex, six edges, six triangles; in white) of the green cell are visualized. (B) Left panel: *P*-values obtained from comparison of *C. hirsuta* versus *A. thaliana* ovules across different stages, taking into account all cells of the ovule. The dashed line indicates *P*=0.05. Right panel: scatter plot of the feature vectors after applying PCA to stage 3-VI ovules of *C. hirsuta* (orange) and *A. thaliana* (blue), taking into account all cells of the analyzed ovule. (C) Left panels: *P*-values obtained from comparison of *C. hirsuta* versus *A. thaliana* ovule tissues across different stages, taking into account all cells of the indicated tissue. The dashed line indicates *P*=0.05. Center panels: scatter plots of the feature vectors after applying PCA to stage 3-VI ovules of *C. hirsuta* and *A. thaliana*, taking into account all cells of the analyzed tissue. Right panels: graphs depicting the coefficient of variation of cell volume (CVcv) for the indicated tissues, stages and species. *C. hirsuta*, orange; *A. thaliana*, blue.

We then asked whether we could detect differences between the two species in the 3D cellular architecture of tissues during development in a rapid and unbiased manner. To address this question, we performed a topological analysis of the 3D cellular architecture of the ovule tissues using the nerve construction, which is a standard tool in topology (Edelsbrunner and Harer, 2010) (Fig. 3A; Materials and Methods). In various fields of mathematics, the nerve is used to encode how a complex geometric object is assembled from simple pieces. Applied to a 3D digital ovule, the nerve captures detailed information about the cellular architecture of the ovule. To evaluate the between-species differences in the nerves we computed, we applied a statistical two-sample test to the ‘feature vectors’ of nerves, as standard statistical tests cannot be applied directly to sets of nerves. Feature vectors are vector representations of the nerves that can be analyzed statistically.

First, we compared the 3D cellular architecture of the entire ovule of *A. thaliana* and *C. hirsuta* (Fig. 3B, Tables S1 and S2). For each developmental stage, we obtained two sets of feature vectors: one set representing *C. hirsuta* ovules and one set representing *A. thaliana* ovules. The feature vectors after applying principal component analysis (PCA) to stage 3-VI ovules and tissues of *C. hirsuta* and *A. thaliana*, taking into account all cells, are shown in Fig. 3B (right panel). We applied the multivariate two-sample test of Baringhaus and Franz to these feature vectors (Baringhaus and Franz, 2004), and obtained *P* values via bootstrapping (Fig. 3B, Tables S1-S3). The *P* values indicated evidence of a species difference in the cellular architecture of the ovule as a whole from stage 2-III, with the exception of stage 3-IV. As a further control, we tested whether the nerve construction could distinguish between the outer integument and the chalaza within each of the two species. For both species and all developmental stages, the resulting *P*-value was, at most, 0.0001 (Table S3), indicating that this approach can detect the expected topological differences between the two tissues within a species, underscoring the value of our topological approach for discriminating the 3D cellular architecture of tissues.

We then asked whether the information about the 3D cellular architecture contained in the nerve can distinguish between homologous tissues of comparable stage of these two species. We investigated the 3D cellular architecture of the main ovule tissues of *A. thaliana* and *C. hirsuta* from stage 2-III to 3-VI. The *P* values indicated evidence for a difference in the cellular architecture of the outer integument, inner integument and chalaza between the two species, beginning at stage 3-I for the outer integument and chalaza, and at stage 3-IV for the inner integument (Fig. 3C, Table S2). We found no statistical support for a difference in cellular architecture for the nucellus and funiculus.

Together, these results suggest that the comparative differences in tissue morphogenesis are not merely related to differences in cell number and volume (Tables S4-S6), but may also be accompanied by differences in the cellular topology of tissues.

### *C. hirsuta* and *A. thaliana* ovules exhibit tissue-specific differences in cell geometry and tissue growth patterns during development

The integuments and chalaza of *C. hirsuta* and *A. thaliana* show differences in shape, as indicated by reduced curvature on the anterior side and the more rounded shape on the posterior side in mature *C. hirsuta* ovules (Fig. 2E,F). We asked whether the observed differences in topology and tissue shape could be related to variations in the 3D cell geometries. After tissue differentiation from stage 2-III onwards, ovule tissues of *C. hirsuta*, with the exception of nucellus and embryo sac, tend to show larger total volumes, cell numbers and cell volumes than corresponding tissues of *A. thaliana* (Fig. S1, Tables S4-S6) (Vijayan et al., 2021). However, a higher cell number or a larger cell volume per se are not the cause of differences in topology. Moreover, the nerve may reveal variations in 3D cell architecture, but does not imply any particular cell parameter. We reasoned that higher variability in cell volume between tissues could affect 3D cellular topology as the differences in the spread of cell volumes might impact the number of neighbors of cells and the neighbors of neighbors of cells, and so on, thereby impacting the topology. Therefore, we compared the coefficient of variation of cell volume (CVcv) for the individual ovule tissues. We found that, in *A. thaliana*, the CVcv for outer integument and chalaza is consistently more than in *C. hirsuta* from stage 3-I (Fig. 3C). The onset of the discrepancy in CVcv for these two tissues correlated with the onset of variation in topology, which also began at stage 3-I (Fig. 3C). We failed to observe CVcv differences for the inner integument and the funiculus. In the case of the inner integument, we therefore concluded that the divergent topologies must have a different cellular basis. In fact, we were able to identify interspecies differences in the way a parenchymal layer develops in the inner integument (see below). As far as the funiculus is concerned, we could not detect any interspecies differences between the results of the topology analysis and when comparing the CVcv values. These findings are consistent with the similar morphology of the funiculi. For the nucellus, we observed inconsistent variability in CVcv values across developmental stages between the two species (Fig. 3C), which did not appear to affect the similarity of nucellar topologies and tissue shape, as suggested by nerve analysis and morphology, respectively.

**Fig. 4.**
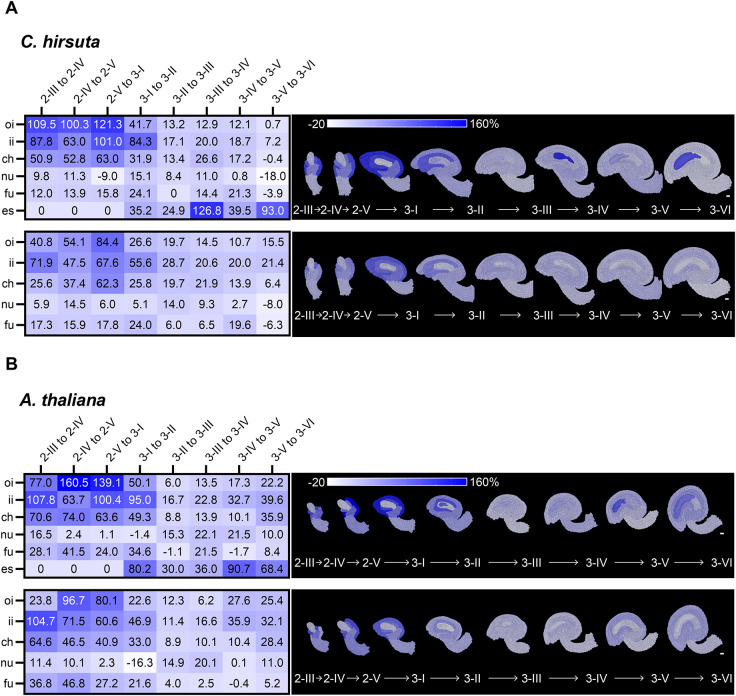
**Comparative trends of relative tissue growth underlying *C. hirsuta* and *A. thaliana* ovule development.** (A,B) *C. hirsuta* (A) and *A. thaliana* (B) relative increase in tissue volume (top) and relative increase in tissue cell number (bottom) between two consecutive stages. (A,B) Heatmaps (left) and mid-sagittal sections (right) depicting relative increase in tissue volume (top) and tissue cell number (bottom) across the different ovule stages from 2-III to 3-VI. Heatmap values indicate the percentage change in mean parameter between stages according to the formula [y(*n*+1) − y(n)]/y(n). Scale bars: 10 µm.

Next, we asked whether the observed variation in topology and cellular parameters in the integuments and chalaza corresponded to tissue-specific differences in growth patterns during their development in *C. hirsuta* and *A. thaliana*. Because we worked with stage-specific cohorts of fixed ovules, we estimated growth patterns indirectly by assessing changes in cell size and cell number between stages. To this end, we took the ratio of the differences between the mean tissue volumes or mean cell numbers of two consecutive stages and the corresponding mean parameter of the previous stage, according to the formula [y(n+1) − y(n)]/y(n). We scored these two relative parameters from stage 2-III to stage 3-VI (Fig. 4A,B).

We observed that, for both species, the outer integument experienced the greatest relative increase in size between consecutive stages from 2-III up to 3-I, followed by a decrease in relative growth in subsequent stages. From stage 2-III to 2-IV, the outer integument of *C. hirsuta* grows 1.4 times more than that of *A. thaliana*, whereas from 2-IV to 2-V, the outer integument of *A. thaliana* grows 1.6 times more than that of *C. hirsuta.* This relative growth is also evident when plotting the relative contribution of outer integument to the total ovule volume (Fig. S2). The relative outer integument proportion continues to increase until stage 3-I, and then stays relatively constant. The relative tissue-specific alterations in cell numbers between stages do not fully explain the relative tissue growth patterns, suggesting that cell growth might play a role. For example, the chalaza of both species shows a consistently higher relative tissue growth than relative increase in average cell number along with a consistent increase in average cell volume over development. However, interestingly, the relative chalaza proportion is higher in *C. hirsuta* than in *A. thaliana* (Fig. S2).

In summary, the combined analysis suggests differences in topology, cellular parameters and stage-wise tissue growth patterns for the outer integument and the chalaza that may explain shape differences between ovules of *C. hirsuta* and *A. thaliana*.

### *C. hirsuta* and *A. thaliana* differ in the formation of the parenchymatic cell layer in the inner integument

Apart from the differences in the inferred overall growth patterns between the inner integuments of *C. hirsuta* and *A. thaliana*, we wondered whether their topological difference was related to variations in the development of their multilayered structure. Starting at stage 3-II, cambium-like activity of the inner (adaxial) layer of the inner integument (ii1, differentiating into the endothelium) in *A. thaliana* generates an additional cell layer (ii1′) located between the ii1 and ii2 layers of the inner integument (Debeaujon et al., 2003; Schneitz et al., 1995; Vijayan et al., 2021). Once formed, the ii1′ layer neither expresses the epidermis-specific *A. thaliana MERISTEM L1* (*ATML1*) nor produces tannins like the ii1/endothelium layer, but remains parenchymal. In *A. thaliana* the ii1′ layer is generated by periclinal cell divisions of only a few scattered ii1 founder cells, followed by anticlinal cell divisions in the ii1′ daughter cells. Eventually, the ii1′ layer forms a ring-like structure covering roughly the proximal half of the inner integument. Formation of this new cell layer in *A. thaliana* exhibits features of layer invasion.

**Fig. 5.**
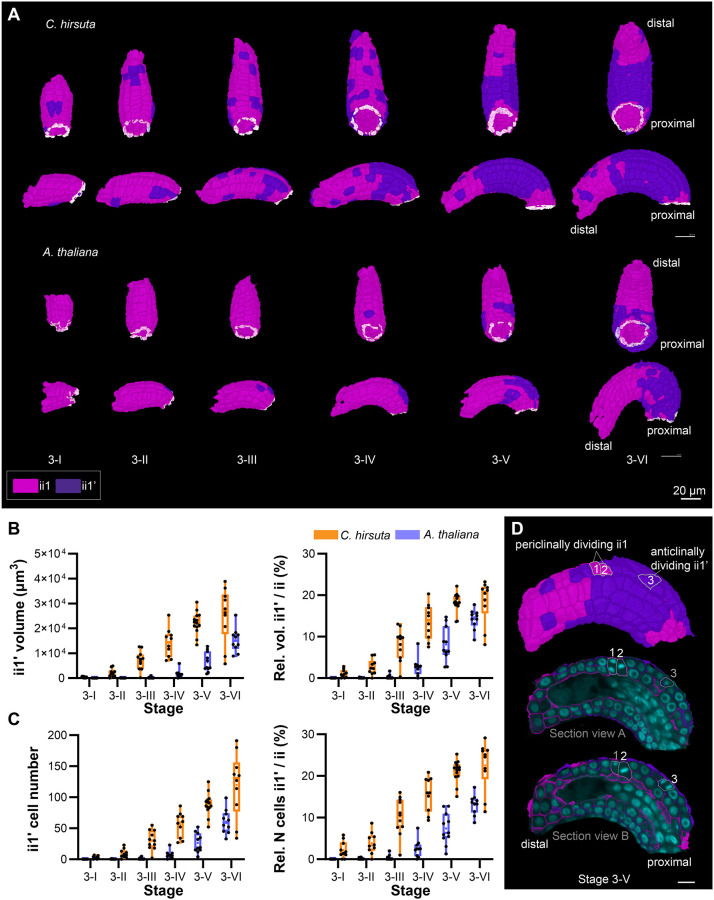
**Comparison of the development of the parenchymatic inner integument layer between *C. hirsuta* and *A. thaliana*.** (A) The origin and propagation of the parenchymatic ii1 (ii1′) layer of *C. hirsuta* and *A. thaliana* ovules from stages 3-I up to 3-VI. Cells of the ii1′ layer are in dark purple. Upper panel: bottom view of 3D surface. Bottom panel: side view of 3D surface. The ii1′ layer emerges as a patch of connected cells but later forms a ring-like structure of connected cells covering the proximal half of the inner integument, but still having patches of cells that are not a part of the ring. (B,C) Quantitative cellular analysis of the ii1′ layer. Data points indicate individual ovules. Data are mean±s.d. Stages are indicated. (B) Left panel: plot depicting the total volume. Right panel: plot depicting the relative contribution of the ii1′ volume to the volume of the entire inner integument. (C) Left panel: total cell number of ii1′. Right panel: the relative contribution of ii1′ total cell number to the cell number of the entire inner integument. (D) Side surface view (top) and section views (middle, bottom) of the 3D cells mesh of a stage 3-V ovule ii1 and ii1′ layers. In the section views, the overlaid nuclei *z*-stack showing the contribution of both periclinal divisions (cells 1 and 2 of ii1) and anticlinal divisions (cell 3 of ii1′) to the propagation of *C. hirsuta* ii1′ layer. Scale bars: 20 µm in A; 10 µm in D.

We could also observe the ii1′ layer in *C. hirsuta* stage 3 ovules (Figs 1B, 5A) and we explored whether there are differences in the formation of this tissue between *C. hirsuta* and *A. thaliana*. In *C. hirsuta*, ii1′ initiation was observed from stage 3-I onward. At stage 3-IV, we also observed the onset of topological variation between the inner integuments of the two species (Fig. 3C). In stage 3-VI ovules, the ii1′ layer not only enveloped the proximal half of the inner integument, but also extended distally, covering more of the inner integument than in *A. thaliana*. In *C. hirsuta*, its distal border was also more irregular, with patches of cells separated from the proximal, more confluent, ii1′ layer (Fig. 5A). We further noticed that the ii1′ layer in *C. hirsuta* occupied a significantly higher proportion of inner integument volume and cell number compared with that in *A. thaliana* (Fig. 5B,C). The increase of the ii1′ layer in terms of both tissue volume and cell number in *C. hirsuta* was double that in *A. thaliana*. Therefore, the ii1′ layer is disproportionately bigger in *C. hirsuta* compared with that in *A. thaliana*.

To understand how the ii1′ layer is initiated and propagated in *C. hirsuta*, we scored 3457 ii1′ cells in addition to all ii1 cells, across the 62 ovules exhibiting an ii1′ layer, for the presence of mitotic divisions. TO-PRO3 staining allows visualization of mitotic figures, which can be used to infer the orientation of the plane of cell division (Fig. 5D) (Vijayan et al., 2021). We observed 70 mitotic divisions in total. These were classified into periclinal ii1 divisions (40/70) and anticlinal ii1′ divisions (30/70). Therefore, 57% of the mitotic divisions could be attributed to periclinal divisions of the ii1 cells. Thirty-four out of 40 (85%) periclinal divisions were observed in stages 3-III to 3-VI. Twenty-five out of the 30 anticlinal divisions were observed in stages 3-V and 3-VI ovules; the remaining five belonged to stages 3-III and 3-IV. These results demonstrate that not only the initiation, but also the propagation of ii1′ in *C. hirsuta* involve periclinal ii1 cell divisions. Whereas, anticlinal divisions can only be attributed toward ii1′ propagation.

Taken together, these results suggest that the disproportionately larger increase of the *C. hirsuta* ii1′ cell layer is due not only to anticlinal cell divisions of ii1′ cells, but also to an approximately equal contribution of periclinal cell divisions in the ii1 layer. This is in contrast to ii1′ growth in *A. thaliana*, in which formation of the ii1′ layer involves a few scattered ii1 periclinal divisions, followed by ii1′ propagation that mostly involves ii1′ anticlinal cell divisions (Vijayan et al., 2021). We further hypothesize that the onset of topological differences in stage 3-IV reflects the dissimilar cellular basis of ii1′ layer development and its proximal-distal extension between *C. hirsuta* and *A. thaliana*.

### Comparative morphometry of the ovule primordia of *C. hirsuta* and *A. thaliana*

Early primordium formation in *A. thaliana* encompasses stages 1-I, 1-II and 2-I (Schneitz et al., 1995; Vijayan et al., 2021). In *A. thaliana*, stage 1 encompasses most of early ovule primordium outgrowth with the onset of expression of a transcriptional reporter for the *WUSCHEL* (*WUS*) gene (pWUS) serving as a convenient marker to delineate non-pWUS-expressing stage 1-I primordia (<50 cells) from pWUS-expressing stage 1-II primordia (>50 cells) (Vijayan et al., 2021). The distinction between stage 1-II and stage 2-I in *A. thaliana* relies on the absence or presence of the MMC (Schneitz et al., 1995). Therefore, in *C. hirsuta*, we also distinguished stage 2-I from stage 1-II ovules based on the presence of the MMC. The upper limit of total volume and cell number of *C. hirsuta* stage 1-II primordia was set based on the similarity in volume to stage 2-I primordia but absence of MMC. To distinguish stage 1-II from stage 1-I in *C. hirsuta*, we extrapolated the *A. thaliana* stage 1-II minimum and maximum values for total volume and cell number to estimate the lower limit of stage 1-II primordia. We used the formula [min_1-II, C. hi._=(min_1-II, A. th._/max_1-II, A. th._)* max_1-II, C. hi_] to set an approximate lower limit of total volume and cell numbers for stage 1-II *C. hirsuta* primordia.

In *C. hirsuta* the maximum volume of stage 1-I primordia is about 1.5×10^4^ µm^3^. The ovule primordia of stage 1-II have a maximum volume of up to 3.7×10^4^ µm^3^ and cell number range from 103-180. Stage 2-I primordia have a volume range of 3.2×10^4^-5.3×10^4^ µm^3^ and cell numbers range from 179-288. We found five out of 15 stage 1-II ovules and three out of 13 stage 2-I ovules with a cell number around 180 and a volume range of ∼3.2×10^4^-3.7×10^4^ µm^3^. These results indicate that the transition from stage 1-II to 2-I in *C. hirsuta* occurs once the primordium has reached about 180 cells and a volume of about 3.5×10^4^ µm^3^, whereas in *A. thaliana* the transition from stage 1-II to 2-I occurs at around 130 cells and a primordium volume of about 1.8×10^4^ µm^3^ (Vijayan et al., 2021). Our data further suggest that ovule primordium outgrowth in *C. hirsuta*, as in *A. thaliana*, is characterized by a steady and continuous stage-wise increase in primordium volume and cell number until late stage 2-I (Fig. S3A). In addition, *C. hirsuta* primordia show slanting (Fig. S3B), which is the first morphological manifestation of an anterior-posterior axis, as observed in *A. thaliana* (Vijayan et al., 2021).

### Planar growth of radial cell layers underlies ovule primordium outgrowth in *C. hirsuta* and *A. thaliana*

The *A. thaliana* ovule follows a general principle of plant organ structure (Satina et al., 1940) and is characterized by three clonally distinct radial layers: the epidermal L1 layer, a sub-epidermal L2 layer and the innermost L3 layer (Jenik and Irish, 2000). In the ovule primordium, the three layers are each made up of one cell layer. Thus, two-dimensional cellular growth within the plane of these radial layers combined with in-plane cell divisions (planar growth) predominantly contributes to primordium formation in *A. thaliana*.

**Fig. 6.**
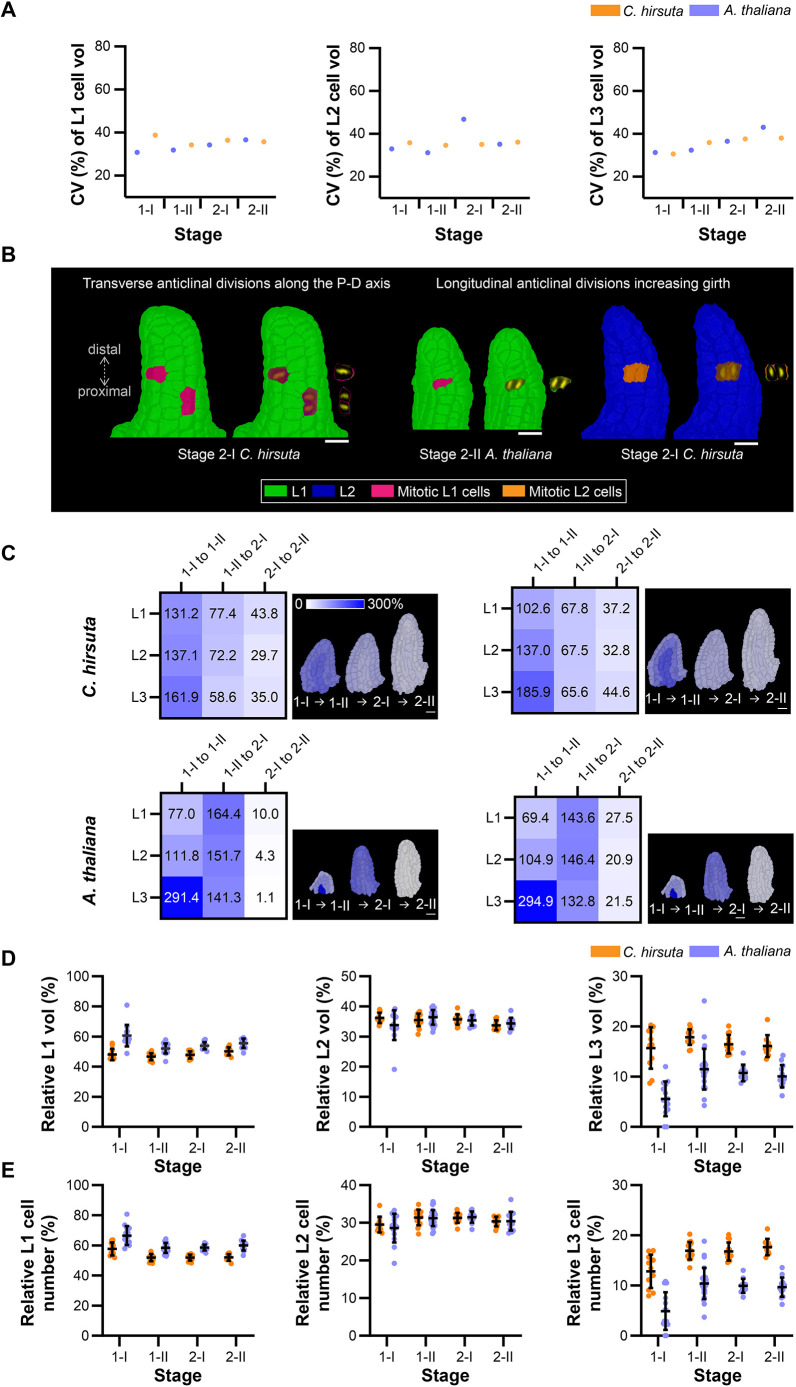
**Differential stage-wise radial layer-specific contributions underlie *C. hirsuta* and *A. thaliana* ovule primordium formation.** (A) Graphs depicting the coefficient of variation of cell volume (CVcv) for the L1 (left), L2 (center) and L3 (right) layers. *C. hirsuta*, orange; *A. thaliana*, blue. (B) 3D cell meshes of the indicated ovule primordia overlaid with TO-PRO3 data for cells showing mitotic figures. Two types of division patterns were inferred from the mitotic figures: transverse anticlinal divisions along the P-D axis and longitudinal anticlinal divisions that increase girth. (C) *C. hirsuta* (top) and *A. thaliana* (bottom) relative increase in tissue volume (left) and relative increase in tissue cell number (right) between two consecutive stages. Heatmaps and mid-sagittal sections depict relative increase in tissue volume (left) and relative increase in tissue cell number (right) across consecutive ovule stages from 1-I to 2-II. Heatmap values indicate % change in mean parameter between consecutive stages according to the formula [y(n+1) − y(n)]/y(n). (D,E) Plots depicting relative contribution of each radial tissue layer to the entire ovule primordium in terms of (D) total volume and (E) total cell number. Data points indicate individual ovule primordia. Data are mean±s.d. Scale bars: 10 µm.

Apart from the obvious difference in size, the ovule primordia of *C. hirsuta* and *A. thaliana* undergo comparable early development (Table 1, Fig. S3) and exhibit a similar cone-like shape until the end of stage 2-I (Fig. 1A,B). Comparative nerve analysis of the primordium as a whole indicated no significant difference in topology through stage 2-II (Fig. 3B, Table S1). Radial layers were also readily recognizable in the *C. hirsuta* ovule primordium (Fig. 1B,C). We hypothesized that a morphologically similar radial layer organization could be reflected in a comparable topology of the cell layers of *C. hirsuta* and *A. thaliana* ovule primordia. Indeed, when we performed a topological analysis on single cell layers across stages 1-1 to 2-II, we did not obtain support for a consistent variation in topology for L1 and L2 (Table S1). However, analysis indicated a clear difference in topology for the L3. Assessing the CVcv values for each layer and stage did not reveal a consistent discrepancy (Fig. 6A). Further morphological examination of the *C. hirsuta* 3D digital ovule primordia suggested that L1 and L2 each consist of a single cell layer, similar to the scenario in *A. thaliana*, but that the L3 appears to have a more complex architecture (Fig. 1B,C). A combination of topological and morphological analysis therefore indicated a similar, but not identical, tissue organization in ovule primordia of the two species.

However, in the absence of a clonal analysis, it remained unclear whether planar growth of individual radial cell layers underlies the outgrowth of the *C. hirsuta* ovule primordium or whether radial layer formation is a consequence of primordium growth. To address this issue, we compared cell division patterns in *C. hirsuta* and *A. thaliana* ovule primordia. We again examined mitotic figures that were visualized using the TO-PRO-3 nuclear stain (Fig. 6B). For example, during the outgrowth of the ovule primordium, preferential anticlinal cell growth (along the radial axis, at right angles to the primordium surface) followed by periclinal cell divisions (parallel to the primordium surface) could lead to the formation of additional radial layers. Periclinal divisions in the absence of prior anticlinal cell growth would result in thinner cells. By contrast, cell growth followed by a transverse anticlinal or longitudinal anticlinal cell division would result in an increase in primordium length along the PD axis or an increase in primordium girth, respectively, but maintain planar growth of a single cell layer.

We analyzed mitotic figures in primordia of stages 1-I to 2-II of both species. A total of 106 primordia comprising 166 mitotic figures and encompassing both species were scored (Table S7). We did not observe a single mitotic figure corresponding to a periclinal cell division, rather all detected mitotic figures indicated either transverse or longitudinal anticlinal cell divisions (Fig. 6B). Because we observed few mitotic figures in L3, we may have missed mitotic figures indicative of periclinal cell divisions in this tissue that could contribute to its more complex architecture in *C. hirsuta*. Nevertheless, our results suggest that the L1 and L2, and possibly the L3, undergo predominantly planar growth that maintains a constant radial layer organization during ovule primordium development not only in *A. thaliana* but also in *C. hirsuta*. It further suggests that the three radial layers each contribute to the increase in ovule primordium size during the developmental period analyzed (Tables 2-4).

**Table 2.**
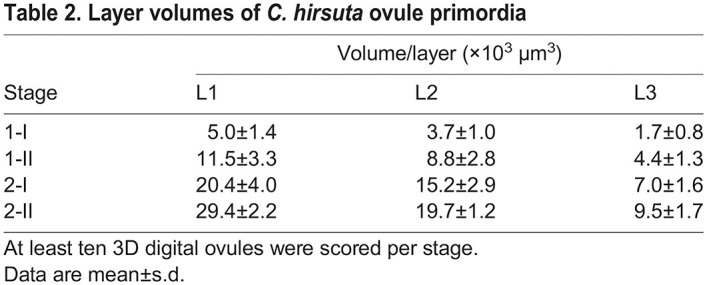
Layer volumes of *C. hirsuta* ovule primordia

**Table 3.**
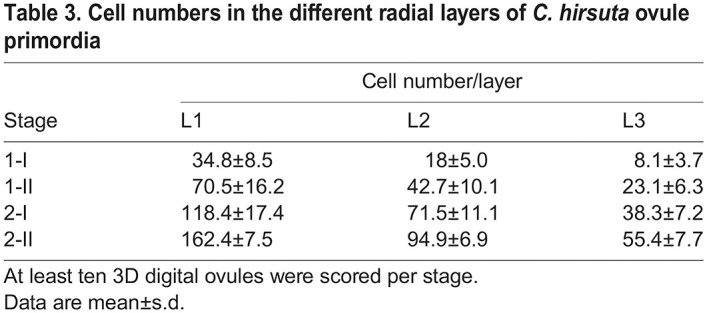
Cell numbers in the different radial layers of *C. hirsuta* ovule primordia

**Table 4.**
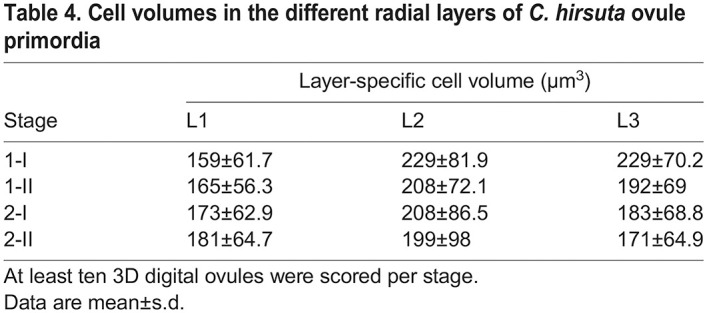
Cell volumes in the different radial layers of *C. hirsuta* ovule primordia

Next, we addressed the relative contributions of cell layer growth to primordium outgrowth by performing a comparative analysis of estimated stage-wise relative layer growth up to stage 2-II, as described above. We observed surprising diversity between *C. hirsuta* and *A. thaliana* (Fig. 6C). Regarding the stage-wise growth of the individual layers, our estimates indicated that in *C. hirsuta*, most relative layer growth occurs during stages 1-I to 1-II, with steadily decreasing layer-specific relative growth during transition from stages 1-II to 2-I and 2-I to 2-II (Fig. 6C). We estimated only slight unequal relative growth of the individual layers. A contrasting picture emerged when we examined the relative layer growth in *A. thaliana* ovule primordia (Fig. 6C). In early stage 1, the volume of the L3 increased primarily, whereas in early stage 2 the L1 dominated and the volume of the L3 increased only minimally. In terms of the relative contribution of each layer to the total primordia volume and cell numbers, the L1>L2>L3 in both species (Fig. 6D,E). However, the L3 contribution is higher in *C. hirsuta*, whereas the L1 contribution is higher in *A. thaliana*. The comparisons indicate a difference in the mechanisms coordinating the relative contributions of the radial layers to ovule primordium outgrowth between the two species.

## DISCUSSION

Here, we provide a reference 3D digital atlas of *C. hirsuta* ovule development at full cellular resolution. First, the atlas provides a valuable resource for future genetic and molecular studies of ovule development in *C. hirsuta*. Second, it adds a high-quality dataset to a growing collection of 3D digital organs that will be of considerable use not only for quantitative single-cell morphometric comparisons of 3D cellular architectures of ovules across plant species, but also for exploring the general properties of complex 3D cell assemblies. Third, the meshes can be used to generate templates for modeling ovule development. As live 3D imaging of ovules at cellular resolution is not currently possible due to their deep burial in the gynoecium, this dataset is based on stage-specific cohorts of fixed samples. Analysis of cellular behavior must therefore rely on estimates based on stage-specific averages of parameters, such as cell size and number.

Digital 3D models can represent complex cell assemblies that are difficult to comprehend through visual analysis of cell morphologies. Topological analysis provides a formal unbiased approach to describe how cells of an organ or tissue are connected in space. It has been used to examine organ design principles in plants with 3D digital organs of *A. thaliana* (Bassel, 2019; Duran-Nebreda et al., 2023; Jackson et al., 2017, 2019). For example, a recent network analysis of several morphologically simple 3D digital organs suggested that the observed 3D cellular architectures at maturity are generated by active patterning mechanisms and do not result from random cell packing processes (Duran-Nebreda et al., 2023).

The ovule is an organ of considerable complexity in terms of its 3D cellular architecture, tissue composition and shape. To identify variations in the 3D cellular architecture of homologous *C. hirsuta* and *A. thaliana* ovule tissues in an unbiased fashion, we applied topological analysis using nerve-based analysis. It represents a robust mathematical approach for studying how complex geometric objects are assembled from simple pieces (Edelsbrunner and Harer, 2010) that, to our knowledge, has not previously been applied to 3D digital organs. As the nerve of a 3D digital ovule is a very complex structure, we computed feature vectors from the nerves that included only local information about the structure around each cell; this was a compromise between the desire to maintain as much information as possible, and the need for a representation of the data that are amenable to statistical analysis. Despite this limitation the results provided leads that could be followed up by quantitative analysis of cell characteristics, inferred tissue growth patterns or visual inspection of cell and tissue morphology.

In terms of abstract cellular structure, i.e. any conceivable way of dividing a given space into abstract cells, the nerve may indicate differences in cellular structure, but it does not suggest any particular cellular parameter. Thus, whether a given parameter of cell geometry correlates with a difference in nerve structure must be determined empirically. We chose the coefficient of variation of cell volume (CVcv) because it measures an aspect of cell structure that is different from the one assessed by nerve analysis. Variation in the CVcv turned out to be associated with differences in cellular architecture between the outer integument and the chalaza of *C. hirsuta* and *A. thaliana*, as suggested by the nerve analysis. Interestingly, in both instances, the small *P*-values from the nerve analysis and the differences in CVcv begin at stage 3-I and the differences in CVcv continue to differ in a similar fashion up to stage 3-VI. In the case of the inner integument, however, there was no noticeable difference in CVcv between the two species and thus no connection between this parameter and the suggested differences in cellular architecture. Nevertheless, closer visual inspection of the cellular basis of ii1′ layer development revealed fundamental differences in this process, resulting in a more extended ii1′ layer in *C. hirsuta* compared with *A. thaliana*. Therefore, for the two integuments and the chalaza, the nerve analysis suggested variations in cellular architecture that could be related to cellular differences.

Inspection of the ovule primordium illustrates the value of a detailed tissue-specific nerve analysis. Nerve analysis of the primordium as a whole did not suggest variation in 3D cellular architecture between *C. hirsuta* and *A. thaliana* ovule primordia, whereas a layer-specific analysis indicated a difference in the innermost L3 layer. Examination of the CVcv did not reveal obvious interspecific differences. However, visual inspection of the L3 layers found that the L3 of *C. hirsuta* was more complex compared with the L3 of *A. thaliana*.

Nerve analysis did not suggest differences in the cellular architectures of the nucellus and the funiculus of *C. hirsuta* and *A. thaliana*. In line with this result, no consistent differences in the CVcv were observed for the funiculus. Moreover, visual inspection did not reveal obvious alterations in its cellular organization. This was not true for the nucellus. The CVcv values fluctuated considerably between stages. Nerve-based analysis also suggested that the nucellus exhibited the highest variability of the feature vectors between samples of a stage-specific cohort compared with other tissues (Fig. S4). Thus, it appears that the cellular architecture of the nucellus is inherently more random than the other ovule tissues from stage 3-I onward, possibly related to the degeneration of three megaspores after meiosis, and to the displacement and crushing of nucellar cells by the developing embryo sac.

Can the results of nerve analysis and cellular examination be related to differences in shape between ovules of the two species? Our results suggest that it depends on the shape and/or the cellular organization of the tissue. In radialized conical structures, such as the ovule primordium and the nucellus, the observed variations in cellular architecture of the innermost layer do not appear to affect the overall shape. In these cases, the outermost layer(s) may constrain the shape, whereas the innermost layer may fill the space. This notion is in line with the role of the epidermis in controlling tissue growth and size (Hacham et al., 2011; Kelly-Bellow et al., 2023; Savaldi-Goldstein et al., 2007). In contrast, the integuments and the chalaza of *C. hirsuta* not only vary in 3D cellular architecture, as indicated by the combined nerve and cellular analyses, but also noticeably differ in shape in comparison with *A. thaliana*. For the integuments, this is indicated by the less pronounced curvature on the anterior side of the *C. hirsuta* ovule. Indeed, the outer integument is known to be a major driver of ovule curvature not only in *A. thaliana* but also in other species (Baker et al., 1997; Endress, 2011; Lora et al., 2011; McAbee et al., 2005; Schneitz et al., 1997; Vijayan et al., 2021). Both integuments are multilayered sheet-like structures with regular cellular organization. This structural design may be inherently more susceptible to shape modification as a consequence of evolutionary shifts in cellular organization. The difference in chalaza shape is indicated by its more bulbous shape at the posterior side of the *C. hirsuta* ovule. Differential growth in the chalaza has been connected to curvature in, for example, *Magnolia grandiflora* (Yamada et al., 2003) and *A. thaliana* (Schneitz et al., 1997; Vijayan et al., 2021). The shape of the mature chalaza in *C. hirsuta* and *A. thaliana* is neither conical nor sheet-like and it has no radial organization comparable with the funiculus. Rather, the cells that make up the chalaza appeared to have undergone spatially irregular cell divisions during chalaza development, which likely contributed to its broadening. Thus, differences between species in the internal cell behavior of the chalaza may provide a plausible explanation for corresponding shape variations. This consideration also underlines the importance of analyzing internal cellular behavior in the context of tissue morphogenesis in plants. It will be interesting to explore the potential relationship between structural tissue design and its susceptibility to shape changes due to modifications in cell behavior in future studies.

## MATERIALS AND METHODS

### Plant work and lines

*C. hirsuta* var. Oxford (Ox), herbarium specimen voucher Hay 1 (OXF) (Hay and Tsiantis, 2006) and *Arabidopsis thaliana* (L.) Heynh. var. Columbia (Col-0) were used as wild-type strains. Plants were grown on soil as described previously (Fulton et al., 2009; Hay et al., 2014).

### Clearing and staining of tissue samples

Treatment of *C. hirsuta* ovules was carried out as described previously (Tofanelli et al., 2019; Vijayan et al., 2021) with some optimizations. Tissue was fixed in 4% paraformaldehyde in PBS for 1.5-2 h followed by one wash in PBS before transfer into the ClearSee solution [xylitol (10%, w/v), sodium deoxycholate (15%, w/v), urea (25%, w/v) in H_2_O] (Kurihara et al., 2015). Clearing was carried out at least overnight or for up to 2-3 days. Cell wall staining with SR2200 (Renaissance Chemicals, Selby, UK) was performed as described previously (Musielak et al., 2015). Cleared tissue was washed in a PBS solution containing 0.1% SR2200 and then put into a PBS solution containing 0.1% SR2200 and a 1/1000 dilution of the nuclear stain TO-PRO-3 iodide (Thermo Fisher Scientific) for 30 min. Tissue was washed in PBS for one minute, transferred again to ClearSee for 20 min before mounting in Vectashield antifade agent (Vector Laboratories).

### Microscopy and image acquisition

Confocal laser scanning microscopy of ovules stained with SR2200 and TO-PRO-3 iodide was performed on an upright Leica TCS SP8 X WLL2 HyVolution 2 (Leica Microsystems) equipped with GaAsP (HyD) detectors and a 63× glycerol objective (HC PL anterior-posteriorO CS2 63x/1.30 GLYC, CORR CS2). Scan speed was at 400 Hz, the pinhole was set to 1 Airy units, line average between 2 and 4, and the digital zoom between 0.75 and 2. For *z*-stacks, 12- or 16-bit images were captured at a slice interval of 0.33 μm with voxel size of 0.126 μm×0.126 μm×0.33 μm. Laser power or gain was adjusted for *z* compensation to obtain an optimal *z*-stack. Image acquisition parameters were the following: SR2200 excitation, 405 nm diode laser (50 mW) with a laser power ranging from 0.1% to 1.5% intensity, detection at 416-476 nm with the gain of the HyD detector set to 20. TO-PRO-3 iodide excitation, white-light laser at 642 nm, with a laser power ranging from 2% to 3.5%, detection at 661 to 795 nm, with the gain of the HyD detector set to 400. Images were adjusted for color and contrast using ImageJ (Rueden et al., 2017) or MorphoGraphX software (Strauss et al., 2022).

### Datasets, 3D cell segmentation and 3D cell meshes

The dataset encompassing the segmented wild-type 3D digital ovules of *A. thaliana* was described earlier (Vijayan et al., 2021). The *z*-stacks of *C. hirsuta* ovules were 3D cell segmented using the PlantSeg pipeline (Wolny et al., 2020). In all instances, cell 3D meshes were generated with MorphoGraphX using segmented image stacks and the process ‘Mesh/Creation/Marching Cube 3D’ with a cube size of 1. Manual cell type labeling was performed with MorphoGraphX.

### Exporting attributes from MorphoGraphX for further quantitative analysis

All quantitative cellular features were exported as attributes from MGX. The attributes included cell IDs (segmentation label of individual cells), cell type IDs (tissue annotation) and cell volume. The attributes from individual ovules were exported as csv files and merged to create long-format Excel sheets listing all the scored attributes of all the cells from the analyzed ovules. Cell IDs with a volume of less than 30 μm^3^ have been excluded from cellular analyses as these correspond to artifacts and empty spaces that are segmented as cells. The files are included in the downloadable datasets.

### Topological analysis

#### The nerve construction

The nerve is closely related to the (unweighted) region adjacency graph, which has been used before to analyze 3D segmentations of plant tissues at cellular resolution (Wolny et al., 2020). This is the graph whose vertices are the cells, and whose edges are the pairs of cells that intersect (i.e. in the setting of 3D digital ovules, pairs of cells that share a voxel). If one models cellular architecture by a simple polyhedral complex (a polyhedral complex in which all polyhedra are simple; for example, the Voronoi tessellation of a generic point set), then the edges in the region adjacency graph correspond to the two-dimensional faces in the polyhedral structure of a cell. However, this graph does not include information about the lower-dimensional faces, which is necessary to reconstruct the entire polyhedral structure. The nerve is a ‘simplicial complex’, a higher-order generalization of a graph, which includes the edges of the region adjacency graph, but also the higher-order intersection information needed to count the lower-dimensional faces of the polyhedral cell. For example, the nerve of a Voronoi tessellation is the Delaunay triangulation. Our analysis does not assume that cells are polyhedra, but in this case it is easier to picture the nerve. To compute the nerve, one thinks of each cell as a vertex, and now computes every set of cells that intersect (i.e. a set *C*_1_,..., *C*_*k*_ of cells intersects if there is a voxel that belongs to *C*_*i*_ for all 1≤*i*≤*k*). The edges of the region adjacency graph are exactly those sets of cells in the nerve that contain two cells. One pictures a set of two cells as an edge, a set of three cells as a triangle and a set of four cells as a tetrahedron (see Fig. 3A); a set of cells with *k* elements is called a (*k*−1)-simplex. Formally, the nerve is a ‘simplicial complex’, which is a generalization of a graph. When we analyze the cellular architecture of a particular tissue in the ovule, we compute the nerve of only those cells in that tissue.

#### Feature vectors

The feature vector of a nerve *N* is defined as follows. The ‘vertex star’ of a vertex *v* of *N* is the set of *k*-simplices of *N* containing *v*, for all *k*≥0. The ‘face-vector’ of *v* is the vector whose *k*-th component is the number of *k*-simplices in the vertex star of *v*. For example, in Fig. 3A, the vertex star of the green cell consists of one vertex, six edges and six triangles, so its face-vector is (1,6,6). The number of 1-simplices in the vertex star of *v* is exactly the number of neighbors of *v*, so this information is included in the face-vector. If a cell in a simple polyhedral complex has face-vector (1,*x*_2_,*x*_3_,*x*_4_), then *x*_2_ is the number of 2-dimensional faces in the polyhedral structure of the cell, *x*_3_ is the number of edges in this structure and *x*_4_ is the number of vertices. To define the feature vector *X* of *N*, we enumerate all possible face-vectors, then let the *i*-th component of *X* be the proportion of vertices of *N* whose face-vector is equal to the *i*-th face-vector in the enumeration. When we compute feature vectors for a set of nerves, it is necessary to enumerate all face-vector vertices of all nerves and to use this enumeration when computing each feature vector.

#### Analysis of differences in topology

We analyzed the difference between species in the 3D cellular architecture of tissues during development in the following way. Fixing a tissue (e.g. the chalaza) and developmental stage, we computed the nerves of the cells of that tissue at that stage. This gives a set *N*_1_,..., *N*_*a*_ of nerves from *A. thaliana*, and a set *M*_1_,..., *M*_*b*_ of nerves from *C. hirsuta*. We computed the feature vectors of these nerves, giving a set *X*_1_,..., *X*_*a*_ of *A. thaliana* feature vectors and a set *Y*_1_,..., *Y*_*b*_ of *C. hirsuta* feature vectors. We applied a multivariate two-sample test (see below) to the vectors *X*_1_,..., *X*_*a*_ and *Y*_1_,..., *Y*_*b*_, and computed the resulting *P*-value. Looking at these *P*-values across developmental stages gives insight into whether there is a difference between species in the 3D cellular architecture of the given tissue, and, if so, at what stage in development this difference becomes visible in the nerve.

### Software

The MorphoGraphX software was used for the generation of cell 3D meshes, cell type labeling and the analysis of 3D cellular features (Barbier de Reuille et al., 2015; Strauss et al., 2022). It can be downloaded from its website (https://morphographx.org). The PlantSeg pipeline (Wolny et al., 2020) was used for 3D cell boundary prediction and segmentation. The software can be obtained from its Github repository (https://github.com/hci-unihd/plant-seg). The C++ code, Python and R scripts, as well as all dependencies required for the topological analysis and its statistical evaluation were packaged using Docker (https://docker.com). The source code and the Dockerfiles can be obtained from the Github repository at https://github.com/fabian-roll/NADO.

### Statistical analysis

Statistical analysis was performed using a combination of R (R Core Team, 2022) with RStudio (RStudio Team, 2020), the Anaconda distribution (Anaconda Software Distribution; https://anaconda.com) of the Python SciPy software stack (Oliphant, 2007) and PRISM10 software (GraphPad Software).

#### Box and whiskers plots

Box plots show the median value of the distribution as a central line and mean value of the distribution as a plus sign within the box. The limits of the box represent the quartiles of the distribution. Whisker ends mark the minimum and maximum of all the data.

#### The multivariate two-sample test

To compare two sets of feature vectors, we use the multivariate two-sample test of Baringhaus and Franz (2004). Given independent random vectors in d-dimensional Euclidean space X1, …, Xm and Y1, …, Yn that are identically distributed with distribution functions F and G, one tests the null hypothesis F=G against the general alternative F ≠ G using a test statistic called the Cramér statistic that is defined using Euclidean distances. Distances between Xs and Ys contribute to the statistic with positive weight, whereas distances between Xs and distances between Ys contribute with negative weight. Rejection of the null hypothesis is for large positive values of the test statistic. Baringhaus and Franz show that *P* values can be estimated using bootstrapping (Van der Vaart and Wellner, 2012). The estimated *P* value can be zero, if the computed value of the test statistic is larger than all the values computed from bootstrap samples. The test is implemented by the R package ‘cramer’ (Franz, 2019).

#### Spread of feature vectors

Given a set of feature vectors, the average of the pairwise Euclidean distances between the vectors is a measure of the spread of that set. This measure of spread is a component of the Baringhaus-Franz two-sample test that was used to compute *P*-values for feature vectors.

## Supplementary Material

10.1242/develop.202590_sup1 Supplementary information
