# Supplementary material for: Topological analysis of 3D digital ovules identifies cellular patterns associated with ovule shape diversity
Source: Development. 2024 May 30;151(20):dev202590. doi: 10.1242/dev.202590 (PMC11168579; doi:10.1242/dev.202590)
Supplement: Supplementary information [file develop-151-202590-s1.pdf]

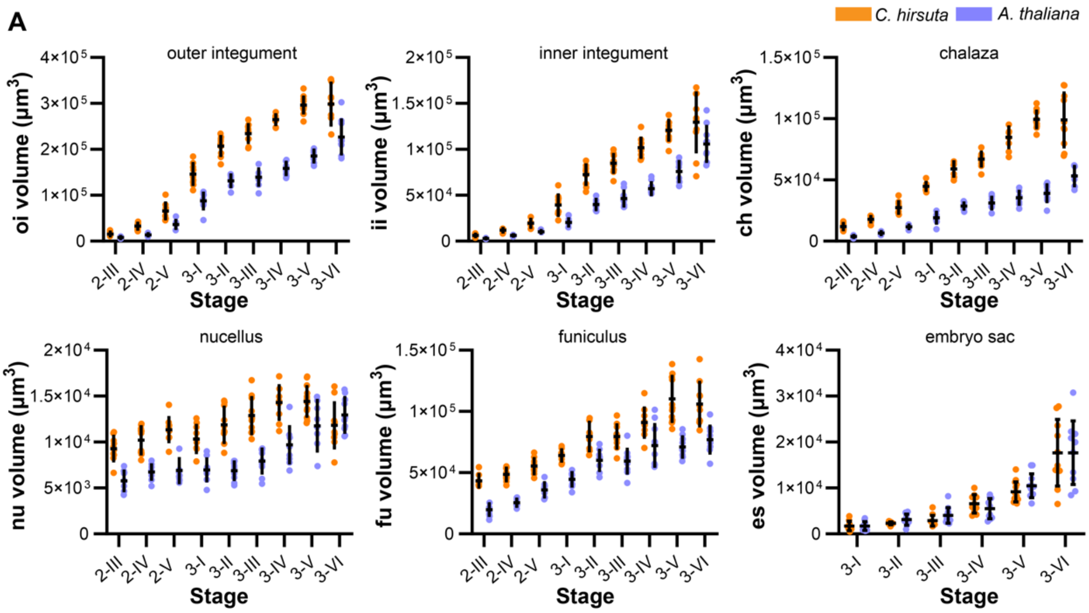

Fig. S1. Comparison of tissue-specific volumes of *C. hirsuta* and *A. thaliana* ovules. Plots comparing total tissue volumes of the outer integument, inner integument, chalaza, nucellus, funiculus, and embryo sac of wild type *C. hirsuta* and *A. thaliana* ovules at stages 2-III to 3-VI. Data points indicate individual ovules. Mean ± SD are represented as bars and whiskers. Abbreviations: ch, chalaza; es, embryo sac; fu, funiculus; ii, inner integument; nu, nucellus; oi, outer integument.

Development • Supplementary information

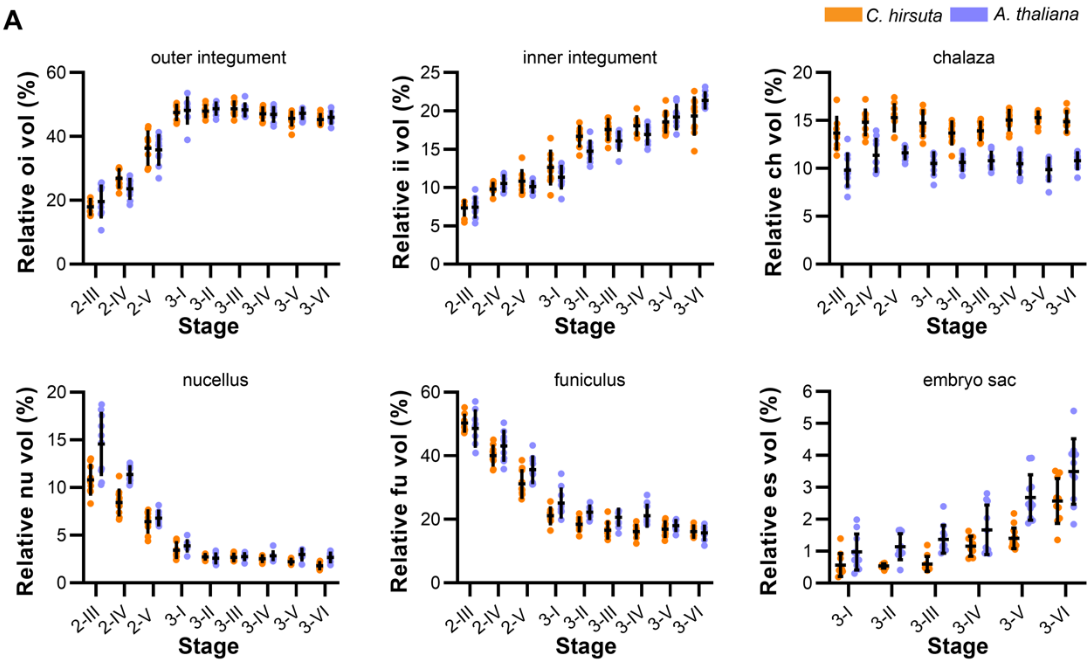

Fig. S2. Comparative differences in relative contributions of tissue-specific volumes to the entire ovule. Plots comparing the relative contribution of the volume of each tissue to that of the entire ovule for both *C. hirsuta* and *A. thaliana* at different stages. Data points indicate individual ovules. Mean ± SD are represented as bars and whiskers. Abbreviations: ch, chalaza; es, embryo sac; fu, funiculus; ii, inner integument; nu, nucellus; oi, outer integument.

Development • Supplementary information

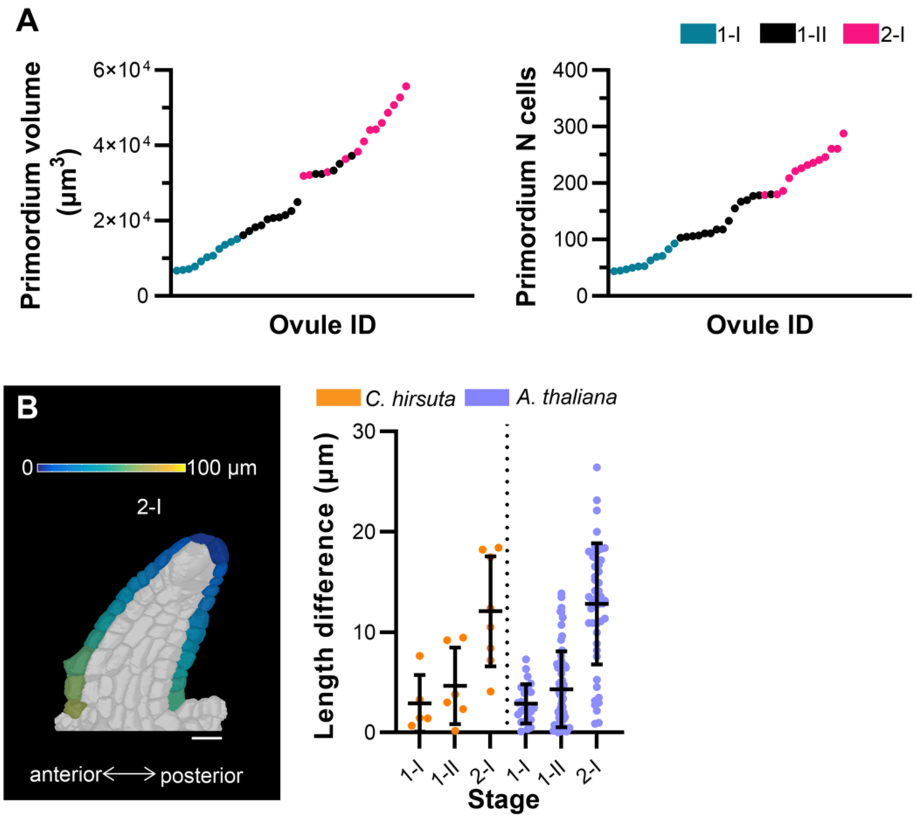

Fig. S3. Developmental features underlying the growth of *C. hirsuta* ovule primordia. (A) *C. hirsuta* primordia grow in a continuous manner. (A) Left panel: plot indicating the total volume of primordia ordered according to increasing volume. Right panel: plot depicting the total number of cells in the ovules ordered according to the increasing number of cells. Data points indicate individual ovules and are colored by stage. (B) C. hirsuta primordia show slanting. Left panel: 2D section view of a stage 2-I 3D cell mesh; the heatmap on the surface cells of posterior and anterior halves depicts the quantified distance value between individual measured cells to the distal tip of primordia. Right panel: plot showing a comparison of the extent of slanting of *C. hirsuta* and *A. thaliana* primordia, quantified by the difference in maximal length on the anterior and posterior sides of ovule at stages 1-I, 1-II, and 2-I. Data points indicate individual ovules. Mean ± SD are represented as bars and whiskers.

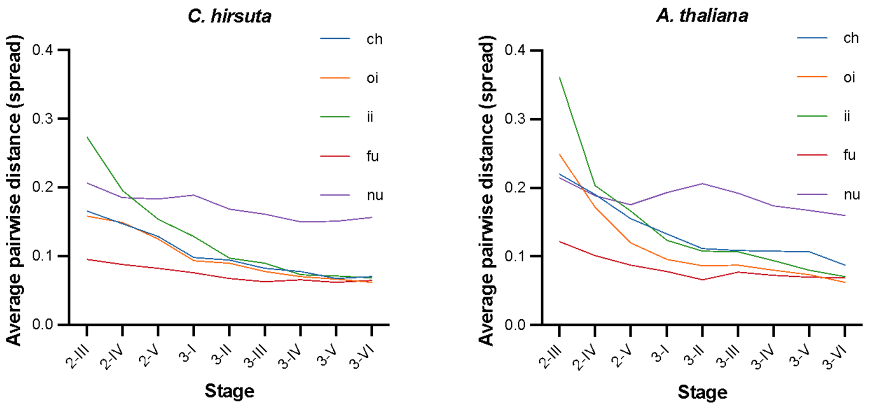

Fig. S4. Spread of the tissue-specific feature vectors of *C. hirsuta* and *A. thaliana* ovules between samples of a given stage-specific cohort. Plots depicting the spread of feature vectors for the specified tissues of *C. hirsuta* (left) and *A. thaliana* (right). Given a set of feature vectors, the average of the pairwise Euclidean distances between the vectors is a measure of the spread of that set. Abbreviations: ch, chalaza; fu, funiculus; ii, inner integument; nu, nucellus; oi, outer integument.

Development • Supplementary information

Table S1. P values from two-sample test applied to nerve-based feature vectors of entire ovule primordia and their radial cell layers comparing *A. thaliana* and *C. hirsuta*

Stage	Tissue			
	Primordium	L1	L2	L3
1-I	0.4	0.067	0.11	-
1-II	0.07	0.0033	0.02	0.0032
2-I	0.22	0.35	0.012	0.0075
2-II	0.19	0.02	0.25	0.0067

≥ 10 3D digital ovules scored per stage.

Table S2. P values from two-sample test applied to nerve-based feature vectors of entire ovules and individual ovule tissues comparing *A. thaliana* and *C. hirsuta*

Stage	Ovule	Outer integument	Inner integument	Chalaza	Nucellus	Funiculus
2-III	1.1 x 10^{-3}	4.6 x 10^{-2}	1.3 x 10^{-2}	9.9 x 10^{-2}	1.6 x 10^{-1}	1.5 x 10^{-1}
2-IV	0	1.3 x 10^{-2}	1.7 x 10^{-1}	2.0 x 10^{-1}	1.3 x 10^{-1}	9.0 x 10^{-2}
2-V	1.8 x 10^{-2}	8.4 x 10^{-2}	3.7 x 10^{-1}	2.0 x 10^{-1}	4.6 x 10^{-1}	1.4 x 10^{-1}
3-I	5.0 x 10^{-4}	7.0 x 10^{-4}	3.2 x 10^{-1}	2.0 x 10^{-2}	3.3 x 10^{-1}	1.2 x 10^{-2}
3-II	0	6.5 x 10^{-3}	1.6 x 10^{-1}	9.5 x 10^{-3}	7.1 x 10^{-2}	3.0 x 10^{-2}
3-III	1.0 x 10^{-4}	7.5 x 10^{-3}	1.1 x 10^{-3}	2.0 x 10^{-4}	8.0 x 10^{-2}	7.5 x 10^{-2}
3-IV	8.3 x 10^{-2}	1.0 x 10^{-3}	4.0 x 10^{-4}	9.6 x 10^{-3}	2.5 x 10^{-1}	1.9 x 10^{-1}
3-V	0	1.0 x 10^{-4}	1.0 x 10^{-4}	1.0 x 10^{-4}	1.3 x 10^{-1}	1.3 x 10^{-2}
3-VI	9.0 x 10^{-4}	6.0 x 10^{-4}	5.2 x 10^{-3}	9.0 x 10^{-4}	2.8 x 10^{-1}	5.6 x 10^{-2}

≥ 10 3D digital ovules scored per stage.

Table S3. P values from two-sample tests applied to nerve-based feature vectors distinguishing the chalaza and outer integument in *A. thaliana* and *C. hirsuta*

Stages	*C. hirsuta*	*A. thaliana*
2-III	0	3.0×10^{-4}
2-IV	0	0
2-V	0	0
3-I	0	0
3-II	0	1.0×10^{-4}
3-III	0	3.0×10^{-4}
3-IV	0	0
3-V	0	0
3-VI	0	0

≥ 10 3D digital ovules scored per stage.

Development • Supplementary information

Table S4. Total volumes of major ovule tissues in *C. hirsuta* and *A. thaliana*

	Tissue volume (x10^4 µm^3)									
	Nucellus		Central region		Inner integument		Outer integument		Funiculus	
Stage	C.hi	A.th.	C.hi	A.th.	C.hi	A.th.	C.hi	A.th.	C.hi	A.th.
2-III	0.9 ± 0.1	0.6 ± 0.1	1.2 ± 0.3	0.4 ± 0.08	0.6 ± 0.1	0.30 ± 0.07	1.6 ± 0.4	0.8 ± 0.2	4.3 ± 0.5	2.0 ± 0.5
2-IV	1.0 ± 0.1	0.7 ± 0.1	1.8 ± 0.2	0.7 ± 0.01	1.2 ± 0.2	0.62 ± 0.08	3.3 ± 0.6	1.4 ± 0.3	4.9 ± 0.5	2.5 ± 0.3
2-V	1.1 ± 0.1	0.7 ± 0.1	2.7 ± 0.5	1.2 ± 0.2	2.0 ± 0.4	1.0 ± 0.1	6.6 ± 1.7	3.4 ± 0.6	5.5 ± 0.6	3.4 ± 0.8
3-I	1.0 ± 0.1	0.7 ± 0.1	4.5 ± 0.3	1.9 ± 0.4	3.9 ± 1.1	2.04 ± 0.4	14.6 ± 2.3	8.8 ± 1.7	6.4 ± 0.5	4.5 ± 0.6
3-II	1.2 ± 0.2	0.7 ± 0.1	5.9 ± 0.6	2.9 ± 0.3	7.2 ± 1.1	4.0 ± 0.6	20.7 ± 2.1	13.2 ± 1.3	8.0 ± 1.0	6.0 ± 0.8
3-III	1.3 ± 0.2	0.8 ± 0.1	6.7 ± 0.6	3.1 ± 0.5	8.5 ± 1.0	4.7 ± 0.8	23.4 ± 2.0	13.9 ± 1.8	8.0 ± 1.0	6.0 ± 1.0
3-IV	1.4 ± 0.2	1.0 ± 0.2	8.5 ± 0.8	3.6 ± 0.5	10.2 ± 1.0	5.8 ± 0.6	26.5 ± 1.1	15.9 ± 1.3	9.1 ± 1.2	7.3 ± 1.7
3-V	1.4 ± 0.2	1.2 ± 0.3	9.9 ± 0.7	3.9 ± 0.7	12.1 ± 1.1	7.6 ± 1.1	29.7 ± 1.7	18.6 ± 1.3	11.0 ± 1.8	7.1 ± 0.8
3-VI	1.2 ± 0.2	1.3 ± 0.2	9.9 ± 2.2	5.3 ± 0.8	13.0 ± 3.2	10.6 ± 1.9	29.9 ± 4.6	22.7 ± 3.7	10.6 ± 1.8	7.7 ± 1.1

C.hi: *C. hirsuta*, A.th: *A. thaliana*
≥ 10 3D digital ovules scored per stage.

Values represent mean ± SD.
Values for *A. thaliana* were obtained from Vijayan et al., 2021.

Table S5. Total cell numbers of major ovule tissues in *C. hirsuta* and *A. thaliana*

	Tissue cell number									
Stage	Nucellus		Central region		Inner integument		Outer integument		Funiculus	
	C.hi	A.th.	C.hi	A.th.	C.hi	A.th.	C.hi	A.th.	C.hi	A.th.
2-III	52.2 ± 6.1	58.1 ± 8.1	72.6 ± 11.7	35.0 ± 5.7	33.8 ± 5.1	23.5 ± 5.5	62.5 ± 12.7	53.8 ± 12.1	223.8 ± 27.1	153.7 ± 28.5
2-IV	55.3 ± 6.9	64.6 ± 5.9	91.2 ± 8.7	57.6 ± 9.7	58.1 ± 8.6	48.1 ± 5.9	88 ± 10.5	66.6 ± 10.6	262.5 ± 24.3	210.2 ± 23.6
2-V	63.3 ± 7.7	64.5 ± 10.5	125.3 ± 20.6	85.0 ± 12.2	85.7 ± 16.7	79.3 ± 8.4	135.6 ± 20.1	124.0 ± 20.2	304.2 ± 34.1	295.9 ± 50.0
3-I	67.1 ± 5.6	66.9 ± 13.4	203.3 ± 20.6	118.9 ± 25.4	143.6 ± 31.5	132.6 ± 18.2	250 ± 30.6	235.9 ± 37.6	358.4 ± 33.4	392.4 ± 36.4
3-II	70.5 ± 6.8	56.0 ± 14.4	255.8 ± 26.3	158.1 ± 16.6	223.5 ± 33.7	194.7 ± 22.9	316.4 ± 27.8	289.1 ± 12.6	444.3 ± 31.5	477.1 ± 54.2
3-III	80.4 ± 11.2	64.4 ± 16.6	306.1 ± 31.9	172.2 ± 23.4	287.6 ± 45.2	216.8 ± 25.4	378.8 ± 35.7	324.5 ± 21.4	471 ± 46.2	496.2 ± 50.6
3-IV	87.8 ± 13.6	77.7 ± 12.2	373 ± 40.3	191.3 ± 20.1	346.8 ± 44.1	255.8 ± 28.4	433.7 ± 33.8	349.6 ± 26.6	501.6 ± 53.6	511.1 ± 75.8
3-V	90.1 ± 13.2	77.4 ± 20.4	424.9 ± 26.9	209.2 ± 31.7	416.1 ± 40.8	343.5 ± 48.3	480.3 ± 16.6	439.8 ± 57.0	600 ± 76.3	506.8 ± 40.1
3-VI	82.9 ± 15.3	85.9 ± 17.2	452.2 ± 92.1	268.5 ± 31.4	505.3 ± 125.5	453.9 ± 69.8	554.7 ± 97.5	551.6 ± 62.7	562 ± 68.0	533.0 ± 68.6

C.hi: *C. hirsuta*, A.th: *A. thaliana*
≥ 10 3D digital ovules scored per stage.
Values represent mean ± SD.
Values for *A. thaliana* were obtained from Vijayan et al., 2021.

Table S6. Cell volumes of major ovule tissues in *C. hirsuta*

	Tissue cell volume (µm³)				
Stage	Nucellus	Central region	Inner integument	Outer integument	Funiculus
2-III	177.7 ± 242.3	163.9 ± 64.5	188.7 ± 84.5	251.8 ± 103.4	193.3 ± 73.46
2-IV	184 ± 235.7	196.9 ± 92.1	206.4 ± 86.1	374.6 ± 163.4	184.4 ± 70.37
2-V	178.7 ± 210.7	219 ± 112.5	227.9 ± 96.9	486.7 ± 238.9	181.1 ± 75.96
3-I	155.7 ± 113.6	225.2 ± 137.4	283.1 ± 131.3	603 ± 345.6	180.4 ± 73.56
3-II	166 ± 117.2	229.4 ± 160.4	323.5 ± 159.5	653.5 ± 405.5	177.7 ± 78.8
3-III	158 ± 104	217.4 ± 166	294.3 ± 153.3	617.7 ± 392.8	167.5 ± 73.5
3-IV	160.7 ± 105	225.5 ± 183.5	293.2 ± 151.8	609.1 ± 391.4	179.7 ± 77.3
3-V	157.6 ± 89.6	231.8 ± 205.5	289.9 ± 145.2	616.4 ± 416	182.3 ± 82.8
3-VI	139.5 ± 102	216.8 ± 200.7	256 ± 132.7	537.5 ± 335.3	187.1 ± 83.5

≥ 10 3D digital ovules scored per stage.
Values represent mean ± SD.

Table S7. Orientation of mitotic figures in *C. hirsuta* and *A. thaliana* relative to the main axes of the ovule primordia

Species	N[a]	N mitotic figures	N transversal anticlinal cell divisions (along PD axis)[b,c]			N longitudinal anticlinal cell divisions (division increasing girth)[d]			Number of periclinal cell divisions (divisions along the radial axis)
C. hirsuta	44	75	69			6			0
			37 (L1)	27 (L2)	5 (L3)	1 (L1)	4 (L2)	1 (L3)	
A. thaliana	62	91	81			10			0
			49 (L1)	29 (L2)	3 (L3)	2 (L1)	7 (L2)	1 (L3)	

[a]Number of primordia.
[b]Stages 1-I to 2-II were analyzed.
[c]Radial layers (L1, L2, L3) are indicated.
[d]Stages 1-1 to 2-II were analyzed. Only stages 2-I and 2-II showed longitudinal anticlinal mitotic figures.
